# Volunteering, health and the homeless – the cost of establishing a student-run primary healthcare clinic serving the inner-city homeless in South Africa

**DOI:** 10.1186/s12913-020-5061-6

**Published:** 2020-03-12

**Authors:** Deanne Johnston, Patricia McInerney, Jacqui Miot

**Affiliations:** 1 Department of Pharmacy and Pharmacology, Faculty of Health Sciences, University of the Witwatersrand, Johannesburg, South Africa; 2 Centre for Health Science Education, Faculty of Health Sciences, University of the Witwatersrand, Johannesburg, South Africa; 3 Health Economics and Epidemiology Research Office, Department of Internal Medicine, School of Clinical Medicine, Faculty of Health Sciences, University of Witwatersrand, Johannesburg, South Africa

**Keywords:** Cost analysis, Establishment, Operational, Student-run clinic

## Abstract

**Background:**

Those who are homeless are more prone to communicable, respiratory and cardiovascular diseases and are less likely to access healthcare services. In South Africa there are no specific public healthcare services tailored to the needs of these communities, particularly if they are immigrants. Trinity Health Services is a student-run inner-city clinic providing free healthcare to the homeless of Johannesburg, South Africa. The clinic operates two nights per month and provides treatment for mainly acute conditions. The purpose of this study was to determine the costs of establishing and operating a student-run clinic for an indigent population.

**Methods:**

This costing analysis used a mixed-methods approach combining an ingredients-based and top-down methodology. The costs, capital and recurrent, pertaining to the establishment and operating of the clinic as well as the cost of treatment per patient were identified and quantified from 1st January 2016 – 31st December 2017.

**Results:**

The capital costs incurred in establishing the clinic were calculated to be £10,968.57 (ZAR 214157.08) and included building alterations, equipment purchased, installations, furniture, application for a pharmacy license, consumables and medications. The recurrent costs per annum were estimated at £17,730.72 (ZAR 346185.54) and comprised of overheads and maintenance, rental, personnel, pharmacy license, consumables and medication. The cost of treatment per patient, included medication dispensed and consumables used in the consultation, was estimated at £3.54 (ZAR 69.05) per visit.

**Conclusions:**

This study summarised the costs of establishing and operating a student-run clinic providing pertinent information essential to the sustainability of the service. It also provides a model for costs associated with free clinics in faith-based and university settings.

## Background

The number of homeless globally was estimated at 100 million in 2005 [1]. The homeless population in South Africa was estimated between 100,000–200,000 in 2008 [2]. Many South Africans come to Johannesburg, the economic hub of the county, in search of employment. A study conducted in 1998 surveyed 7456 people who were homeless in the inner-city with the majority residing on the streets and pavements [3]. These numbers are most likely to have increased with the decline in employment rates [4], increasing population and the ever-increasing cost of living.

In South Africa, social determinants relating to housing, water and sanitation, nutrition, alcohol and substance abuse as well as social cohesion, lead to premature mortality [5]. These social determinants have perhaps the greatest impact on these vulnerable population groups such as the homeless. Furthermore, there are no specialised healthcare services for the homeless provided by the state. This gap is often filled by non-profit organisations such as the Usizo Lwethu Clinic [6] in Durban and MES Impilo Clinic [7] in Johannesburg.

Seager and Tamasane [8] assessed the health characteristics and access to health care of 1247 homeless adults and children in Gauteng, Mpumalanga, Limpopo and the Western Cape. Their study identified sexual assault, sexually transmitted infections, tuberculosis, human immunodeficiency virus, skin diseases, pregnancy and malnutrition as prominent health issues and more than 20% of the adult respondents reported drug and alcohol abuse. The barriers to access to healthcare faced by the homeless included long waiting times and inability to provide proof of residence and/or identification documentation [9] as well as discrimination by staff [8].

In South Africa there is a fragmented healthcare system where approximately only 17% of the population is covered by private medical insurance schemes [10]. The resource limited public healthcare sector provides care for the vast majority of the population. In 2015/2016 the healthcare bill provided for R3 332.00 to be spent per person using public healthcare services [11]. However, there is a move to radically transform the healthcare sector through the implementation of a national health insurance (NHI) policy. The NHI aims to provide equitable, affordable and universal coverage to all South Africans [12]. Primary healthcare forms the foundation of such a system which requires substantial funding and resources to provide cost-effective care for all. There is, too, a shift from curative to preventative models of care in bringing these services to communities.

There is no information relating to the morbidity and mortality amongst the homeless in South Africa. Several studies have shown mortality and morbidity rates to be higher in the homeless [13–15]**.** Homelessness itself is an independent risk factor for mortality for certain conditions, however mortality rates were higher in the homeless in those with substance abuse disorders and infectious diseases [16]. Medical students have responded to the need for additional healthcare services through the establishment of student-run clinics (SRCs). SRCs provide healthcare services where health sciences students take the leading role supervised by professional staff. There are growing numbers of SRCs globally. Simpson and Long [17] surveyed 111 SRCs in 49 Medical Schools in the United States. In Canada there are eight SRCs providing primary healthcare services [18], while in South Africa there are five SRCs associated with Medical Schools [19]. There are different models of SRCs and these vary considerably according to the location of the clinic, services provided and costing of treatment (Table 1).

**Table 1:** Various models of SRCs

**Location**	**Within shelter/facility** E.g. Project Light [20]	**Community based** E.g. University of Saskatchewan SRC [21]	**Mobile** E.g. Students’ Health and Welfare Centers Organization [22]
**Services**	**Interprofessional team approach** E.g. Student Wellness Initiative Toward Community Health [18]	**Specialized services** E.g. Hands of hope [23]	
**Cost of treatment**	**Free service** E.g. Community Aid, Relief, Education, and Support (C.A.R.E.S.) clinic [24]	**Paid by patient** E.g. Chester Community Physical Therapy Clinic [25]	

The services provided by SRCs are supported by the communities they serve, although the number of patients served in such facilities is often unknown. Patients report to be satisfied with the services provided [26–28]. While studies have shown that SRCs are valued by both students and patients very little is known of the cost of such services or the cost impact to the healthcare system. The number of SRCs in the United States doubled in less than 10 years (Smith, 2014), thus the need identify and quantify the costs involved in establishing and managing this service. The various models of SRCs (Table 1) further add to the complexity of the costs involved. This study provides pertinent costing information to individuals and institutions considering establishing a volunteer clinic as well as identifying factors that would need to be addressed in order to ensure the continued sustainability of such a service. Thus both economic and financial costs as well as capital and operations costs were important to include.

### Study setting

Trinity Health Services (THS) is a student-run clinic established in 2004 and is a partnership between the Faculty of Health Sciences, University of the Witwatersrand, and an inner-city church. The church is a place of refuge for homeless people residing in the city and provides daily soup kitchens. The clinic was started when two medical students saw an opportunity to provide basic first aid assistance to people attending the soup kitchen at the church. Soon the services expanded to provide acute primary healthcare and the need for a pharmacy to dispense medication was identified. The clinic was closed from 2011 to 2015 until a community pharmacy license was granted and reopened in 2016. THS operates on alternate Monday nights providing healthcare and treatment for people with mainly acute conditions. Those needing further assistance are referred to nearby public healthcare facilities. On average there have been 271 patient visits per year since the clinic reopened in 2016. The clinic not only benefits the homeless community through the provision of free healthcare services but is valued by student volunteers as an opportunity to serve the community while applying skills and knowledge learnt. THS is self-funded and is dependent on donations received from individuals and companies. The financial sustainability of the clinic is a concern as the community is growing more dependent on the services it provides. Understanding the costs of operating this type of clinic is an essential component towards ensuring financial sustainability of the service. The financial sustainability would also include ensuring ongoing supply of volunteer students, accessing affordable medications and consumables, managing increasing patient numbers and determining the services the clinic can afford to offer. The startup costs, including the cost of donated goods and services, offer useful information to organisations that are considering establishing a similar service.

Therefore, the research questions for this study were:

- *What were the costs which contributed to the establishment and operating of a SRC?*
- *What was the cost of treatment per patient in a SRC?*

## Methods

The design of the study was a retrospective cost analysis which estimated the costs incurred when the clinic was established as well as the ongoing costs of operating the clinic as well as the cost of treatment per patient. The costing analysis used a mixed-methods approach combining an ingredients-based with a top-down methodology where appropriate.

The study period was from 1st January 2016 to 11th December 2017. The costing year was set at 2018 and costs outside that period were adjusted accordingly using the annual average Consumer Price Index [29]. South African Rands (ZAR) were converted to Pounds (£) using an exchange rate of 0.051 [30] (XE Currency Converter, 2018).

The costs pertaining to the establishment and operating of a SRC as well as the cost of treatment per patient were identified, quantified and valued. Costs were divided into capital and recurrent costs as per Table 2. Capital costs were costs incurred in establishing the clinic for instance building alterations. Recurrent costs refer to operating costs and were calculated per annum, for example consumables used. The costs were further divided into financial and economic costs. Financial costs refer to costs which the clinic incurred and paid for, such as medication. This is compared to economic costs which refer to costs that are valued, however, were not paid for by THS, for example donations of equipment. An Equivalent Annual Cost (EAC) for capital items was calculated at an interest rate of 5% based on recommendations from the South African Pharmacoeconomic Guidelines. It was assumed that the renovations, installations and license would have a life of 20 years, whereas furniture and equipment will have a life of 5 years. The upfront cost of stocking the medicines was not annualized due to the recurrent costs of maintaining the stock.

**Table 2:** Description of capital and recurrent costs

Capital costs	Recurrent costs
**Building:** *Description:* Area used by the clinic which includes the consultations rooms, pharmacy and waiting area *Costing method:* Proportion of floor space used *Valuation method:* Rental fees	**Consumables:** *Description:* Any items used in the consultation process that is neither a pharmaceutical or re-usable item (e.g. disposable gloves) *Costing method:* Per patient consumed quantity *Valuation method:* Expenditure records
**Equipment:** *Description:* Equipment used in the by the clinic in the consultation rooms (glucometers, ECG machine) and pharmacy (e.g. dispensing system, temperature monitoring system, electronic balance) *Costing method:* Ingredient based *Valuation method:* Replacement and contract prices	**Medicines:** *Description:* Medicines which are used/administered in the consultation or dispensed to the patient to take home *Costing method:* Shared proportion of patients Not shared-quantity consumed *Valuation method:* Expenditure records
**Furniture:** *Description:* Includes tables, chairs, cabinets, shelving, beds etc. *Costing method:* Proportion of lifetime use *Valuation method:* Replacement and contract price, rental fees	**Overheads and maintenance:** *Description:* Overhead costs include electricity, telephone, internet and stationary. Maintenance costs include pest control, fire extinguishers, waste disposal and house keeping *Costing method:* Proportion of personnel time, proportion of floor space *Valuation method:* Expenditure records
**Consumables:** *Description:* The cost of consumables purchased in stocking the consultation rooms and store rooms for the first-time *Costing method:* Ingredient based *Valuation method:* Expenditure records	**Personnel:** *Description:* Costs relating to supervising doctors and pharmacists as well as student volunteers *Costing method:* Locum rates for students and professional volunteers (doctors, nurses and pharmacists) *Valuation method:* Gross salary
**Medicines:** *Description:* Medicines ordered to stock the pharmacy for the first-time. *Costing method:* Ingredient based *Valuation method:* Expenditure records	

Descriptive statistics, illustrated using graphs and tables, were used to describe the costs obtained. Sources of data included procurement records, service agreements, patients’ records and pharmacy prescriptions, architectural drawings and asset reports. Costs were captured and analysed using Microsoft Excel® 2010.

Ethical approval was obtained from the Human Research Ethics Committee of the University of the Witwatersrand (M170953). Pharmacy prescription records were assigned study numbers and the list of study numbers and patient names were kept in a locked cupboard and only accessible to the researchers.

## Results

### Capital and recurrent costs

The costs of establishing and operating the clinic were identified and valued. The capital costs (i.e. cost of establishing the clinic) were £10,968.57 (ZAR 214157.08) (Table 3a). The recurrent costs (i.e. cost of operating the clinic) were calculated per annum and amounted to £17,730.72 (ZAR 346185.54) (Table 3b). The economic and financial costs are reported in Fig. 2.

**Table 3:** Summary of (a) capital and (b) recurrent costs

		Pharmacy		Clinic		Total	
(a)							
Capital	Building	£985.41	ZAR 19239.73	£1377.17	ZAR 26888.72	£2362.58	ZAR 46128.45
	Equipment	£1601.19	ZAR 31262.63	£862.93	ZAR 16848.37	£2464.12	ZAR 48111.00
	Installation costs	£2470.87	ZAR 48242.78			£2470.87	ZAR 48242.78
	Furniture	£123.95	ZAR 2420.00	£1318.95	ZAR 25752.00	£1442.90	ZAR 28172.00
	Pharmacy licensing	£826.99	ZAR 16146.71			£826.99	ZAR 16146.71
	Consumables	£58.92	ZAR 1150.40	£810.24	ZAR 15819.67	£869.16	ZAR 16970.07
	Medicines	£531.95	ZAR 10386.07			£531.95	ZAR 10386.07
	Total Capital Costs					£10,968.57	ZAR 214157.08
(b)							
Recurrent (annual)	Overheads & Maintenance					£4194.44	ZAR 81894.77
	Rental	£2700.79	ZAR 52731.84	£2251.72	ZAR 43964.04	£4952.51	ZAR 96695.88
	Personnel	£2291.88	ZAR 44748.00	£4842.35	ZAR 94545.00	£7134.23	ZAR 139293.00
	Pharmacy licensing	£184.58	ZAR 3603.88			£184.58	ZAR 3603.88
	Consumables	£108.99	ZAR 2127.90	£478.04	ZAR 9333.56	£587.03	ZAR 11461.46
	Medicines (incl labels and packaging)	£677.94	ZAR 13236.55			£677.94	ZAR 13236.55
	Total Recurrent Costs					£17,730.72	ZAR 346185.54

### Capital costs

The capital costs included the building alterations and installation costs, purchasing of furniture, equipment, consumables and medications as well as the pharmacy licensing fees.

#### Building alterations

The church provides a permanent space for the clinic and pharmacy. The clinic consists of three consultation rooms and a storage room. The consultation rooms were fitted with wash-hand basins including hot water totaling £ 895.34 (ZAR 17481.21). The storage room, used to keep additional consumable materials and equipment, was fitted with fixed shelving amounting to £ 481.83 (ZAR 481.83).

The alterations to the pharmacy were more extensive to meet the minimum requirements to be registered as a community pharmacy at a cost of £ 985.41 (ZAR 19239.73) (Table 3a). The alterations included adding an additional entrance for receiving stock, separate hand basin and sink with cold and hot running water, fixed counters for compounding and dispensing of prescriptions as well as storage of medications.

#### Equipment

The total cost of equipment was £ 2464.12 (ZAR 48111.00) (Table 3a). Equipment purchased for the consultation rooms, costing £ 653.66 (ZAR 16848.37) (Table 3a) included point of care test machines, thermometers, ear, nose and throat diagnostic sets and blood pressure meters. The equipment for the pharmacy was guided once again by the legislative requirements and cost £ 1601.19 (ZAR 31262.63) (Table 3a). It included reference materials (£ 940.22, ZAR 18357.49), dispensing and compounding equipment (£ 133.48, ZAR 2606.14), portable air-conditioner (£ 230.43, ZAR 4499.00) and a refrigerator (£ 245.84, ZAR 4800.00).

#### Installation costs

Installation costs, £ 2470.87 (ZAR 48242.78) (Table 3a), included the temperature monitoring system (£ 38.41, ZAR 750.00) and computerized dispensing system (£ 2432.46, ZAR 47492.78).

#### Furniture

The cost of furniture was £1442.90 (ZAR 28172.00) (Table 3a). Furniture for the consultation rooms included examination beds, desks, chairs, storage cupboards and light stands. Trestle tables, chairs and curtain dividers were purchased for the waiting area. Less furniture was needed for the pharmacy and included chairs and shelving.

#### Pharmacy licensing

The cost of the pharmacy license, £ 826.99 (ZAR 16146.71) (Table 3a), was a considerable expense in establishing the pharmacy. This cost included pharmacy premise’s application for licensing paid to the National Department of Health of South Africa while the Recording of New Pharmacy, Recording of Pharmacy Owner and Application for Registration of the Responsible Pharmacist were fees paid to the South African Pharmacy Council. The Responsible Pharmacist, appointed by the owner, is a registered pharmacist with defined duties and responsibilities to ensure the pharmacy complies with applicable legislation.

#### Consumables

Consumables needed to stock the consultation and storage rooms were purchased initially to ensure services could be provided. The consumable items purchased were costed at £ 869.16 (ZAR 16970.07) (Table 3a). The clinic established minimum stock levels for consumable items based on projected patient numbers per month. Stationery purchased at £ 58.92 (ZAR 1150.40) also contributed to the cost of consumables.

#### Medication

As per consumables purchased, a once-off consignment of medication was purchased according to the clinics’ formulary to initially stock the pharmacy. This holding stock was considered a capital cost, thereafter the replenishment of medicines was attributed to the operating costs of the clinic. The clinic purchases medication at the single-exit price (SEP) through a wholesaler. The SEP is the regulated price at which a medication may be sold at in the private sector in South Africa. The clinics formulary consisting of 53 items, was in alignment with Standard Treatment Guidelines and the Essential Medicines List of South Africa [31]. It also includes additional medications as requested by prescribers in consultation with the pharmacist. Minimum quantities for medication were established and amounted to £ 531.95 (ZAR 10386.07) (Table 3a).

The total cost of medication is however dependent on several factors which include the availability of active ingredients needed to manufacture the medicine, the cost of originator versus the generic medicine and the additional cost of medication if packed in patient-ready packs or blisters packs.

### Recurrent costs

#### Overheads and maintenance

The costs of overheads and maintenance were £1718.35 (ZAR 33550.11) and £24 76.09 (ZAR 48344.66). Costs contributing to the overheads were electricity, water, security and internet. Maintenance costs included pest control, servicing of the fire extinguishers, calibration of the pharmacy scale, disposal of pharmaceutical and medical waste as well as services fees for the temperature monitoring and dispensing systems.

#### Rental

Monthly rental in the area the church is located area is approximately £5.01/m^2^ (ZAR 98.00/m^2^). The rental cost of permanent space i.e. pharmacy (44.84m^2^), consultation (22.52m^2^) and storage (7.02m^2^) rooms, was £4098.76 (ZAR 80026.76). The rental of shared space, the waiting area, was portioned according to monthly usage at a cost of £472.48 (ZAR 9225.00) per annum.

The church allows the clinic to use this space at no cost. This is an economic cost and contributes 29.7% to the overall annual operating cost.

#### Personnel

The clinic relies on volunteers, both qualified professionals and students, to form the workforce of the clinic. Although all personnel were volunteers, their cost was calculated according to hourly locum rates. The annual cost of pharmacy staff was calculated as £22 91.88 (ZAR 44780.00) for a pharmacist and five pharmacy assistants. There were two medical doctors and ten undergraduate medical students working each evening and calculated costs were £4842.35 (ZAR 94545.00) per annum.

The cost of personnel was accounted for during the times the clinic was operational, however administrative duties that staff, and students complete at other times were not taken into consideration.

#### Pharmacy licensing

Following the initial registration fees for the pharmacy license, annual fees (£184.58, ZAR 3603.88) for the pharmacy and responsible pharmacist were paid to the regulatory authority. All pharmacy personnel paid annual registration fees however these were not included.

#### Consumables

The cost of consumables was £ 587.03 (ZAR 11461.46) and included stationery and disposable items used in the consultations. The exact cost of consumables used in the consultations could not be determined from patient records. Therefore, the cost was estimated based on average of 12 consultations per/night and provided a minimum cost of consumables used which included gloves, masks and linen savers.

#### Medicines

A total of 573 prescriptions were reviewed from 2016 and 2017, consisting of 1054 items dispensed. The cost of medication dispensed in 2016 and 2017 was £394.20 (ZAR 7555.58) and £981.32 (ZAR 18808.65) respectively. The average cost of medicines for the two-year period was calculated (including the cost labelling and packaging) as £677.94 (ZAR 13263.55) (Table 3b) and contributed 3.8% to the total operating costs.

**Fig. 1:**
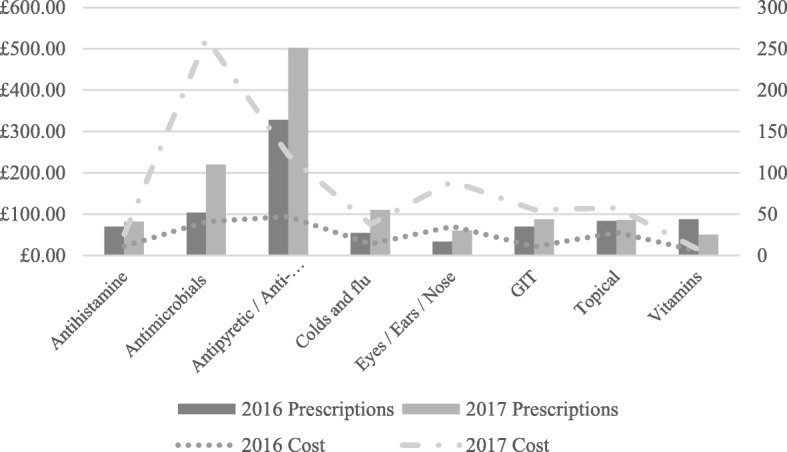
Cost and quantity medication dispensed in 2016 and 2017 (GIT: gastrointestinal tract)

Figure 1 groups medication and depicts expenditure and the number of prescriptions. There was a marked increase in the antimicrobials and antipyretic/anti-inflammatory medications dispensed with a correlating increase in cost. Paracetamol 500 mg tablets (£82.16, ZAR 1611.02; prescriptions: 132), framycetin/gramicidin/dexamethasone eye drops (£52.37, ZAR 1026.85; prescriptions: 6), and cefuroxime 500 mg tablets (£32.97, ZAR 646.50; prescriptions: 3) were the highest contributors to the total costs of medication in 2016 as compared to amoxicillin/clavulanate 1000 mg tablets (£215.41, ZAR 4223.68; prescriptions: 25), paracetamol 500 mg tablets (£ 128.82, ZAR 2525.79; prescriptions: 174) and cefuroxime 500 mg tablets (£87.91, ZAR 1724.00; prescriptions: 8) in 2017.

### Running cost of treatment per patient

The average running cost of treatment per patient per visit was £3.53 (ZAR 69.05) and comprised of medication (£2.36, ZAR46.36) and consumables used in the consultation (£1.18, ZAR 22.69). It represents the financial cost incurred by the clinic in treating a patient per visit. All the additional recurrent costs are donated either by the church or through volunteer services. If these are included in the running costs, the total cost per visit is £61.73 (ZAR 1210.43). If the annualized cost of the capital items (£319.02; ZAR 6255.27) is included, this would add an additional £1.12 (ZAR 21.87) per visit. If both financial and economic capital and operating costs were included, the total cost per consultation would be £62.85 (ZAR 1232.31).

### Financial versus economic costs

**Fig. 2:**
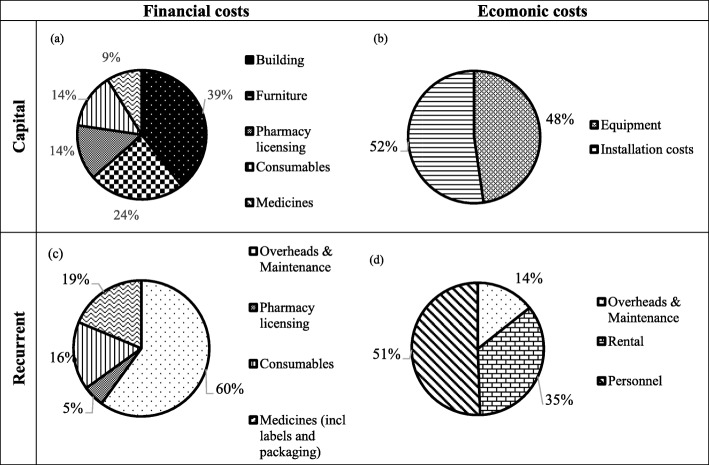
Capital and recurrent costs itemized as financial and economic costs

The analysis of the financial and economic costs, Fig. 2, distributes the costs described in Table 2. The majority of costs, 66%, were economic costs. The salaries of personnel and rental costs were the highest in this group.

Costs relating to building alternations, equipment and installation costs contributed the most to capital costs (65%). Equipment and installation costs were donations the clinic received. Therefore, building alterations and furniture, contributing 62% to the financial costs, were the most expensive capital costs incurred.

The pharmacy license directly contributed 7% of the capital costs. There were numerous costs incurred for the renovations, equipment and installation costs required to meet the minimum standards for a community pharmacy as set out in Pharmacy Act 53 of 1974 of South Africa [32]. The minimum standards include a computerised dispensing and temperature monitoring system, contributing 22% of the capital costs.

The costs relating to personnel and rent contribute 68% of the recurrent costs yet both of these are economic costs. The maintenance costs of the computerized dispensing and temperature monitoring system were donations received. Therefore, the financial costs relating to overheads and maintenance were £ 2163.63 (ZAR 42424.17) and contributed 60% of the financial recurrent costs. The cost of medication (19%) and consumables (16%) were substantial financial costs incurred.

## Discussion

This study provided an in-depth cost analysis of a student-run clinic in South Africa and serves as model which could be of value in the establishment of other SRCs and free clinics. The results pertaining to the recurrent costs are of intrinsic value to THS in determining the feasibility of the services provided.

The clinic fundraises to cover costs of medication and consumables, waste disposal as well as annual pharmacy license fees. To ensure the financial viability of THS both the financial and economic costs need to be considered, ensuring there are sufficient funds are raised to cover all costs and not only the current expenses. This study has helped THS develop short- and medium-term budgets contributing to the model of sustainability. If the annualized capital costs and recurrent donated costs and services are taken into consideration, the total cost per visit is high at £62.85 (ZAR1232.31) compared to the actual running cost £3.53(ZAR 69.05) currently incurred by THS in supplying these services to the patients attending this clinic. This is a reflection of the fact that the clinic only operates twice a month, if it were to operate more frequently and increase the number of visits, the total cost per visit would be reduced. However, this could result in a reduction in willingness to provide volunteer or donated services. A balance is needed between these economic and financial factors to ensure the sustainability of the THS clinic.

### Findings in relation of other studies

The costs associated with establishing a SRC was not found in the literature. Though little information of the operating costs is available, details of the costs incurred were lacking. A survey conducted in free clinics (2005/2006) in the United States, including SRCs, determined a mean operating budget in 748 clinics of $287,810.00/annum [33]. The median operating budget in SRCs in the United States was $12,000.00 ($500.00–95,000.00) per annum of which most sources of funding were private and community grants or through student fundraising [17]. A lack of funding has been identified as a major challenge facing no fee SRCs [34].

The clinic relies on the altruistic nature of volunteers, both students and professional staff, to enable the clinic to function. The cost of personnel contributed 40% to the recurrent costs. The cost of personnel in primary healthcare forms a substantial component of the budget. The cost drivers in an HIV-infected Adult Clinic in South Africa were human resources and antiretrovirals, each contributing a third of the total costs [35]. In Ghana personnel accounted for 60% of the expenditure in Health Centers and Community-based Health Planning and Services [36]. The difference between the cost of personnel between studies could be attributed to the operational hours of the services. THS operates every fortnight and the analysis was based on the number of hours worked, if THS were open more often the cost of personnel would escalate.

The cost of treatment per patient provided an overview of the costs incurred. The cost incurred in accordance to a specific diagnosis could not be calculated with the data available. The majority of the local studies found in the literature provide a cost pertaining to specific conditions for example human immunodeficiency virus [37] and tuberculosis [38]. A study conducted in 2000/1 at a rural primary healthcare clinic in KwaZulu-Natal found the cost of treatment varied substantially from ZAR37.38 (chronic care) to ZAR87.94 (mental health) depending on the diagnosis and severity of the condition [39]. The cost per treatment per patient provided in this study is of intrinsic value to the clinic when budgeting for patient care. However, including the diagnosis when gathering data is imperative for future studies as well as comparing treatment costs in various settings.

Medication purchased contributed 9% to the capital and 19% to the recurrent costs. The Columbia-Harlem Homeless Medical Project found the greatest expenditure in their budget was medication [40]. THS through implementation of a clinic formulary attempts to reduce medication costs. Fee-free clinics elsewhere often dispense free samples or donations provided by pharmaceutical manufacturers [33], however, the Medicines and Related Substances Act 101 of 1965 [41] in South Africa prohibits any donations and sampling of scheduled medicines.

SRCs are often linked to religious based organizations [42, 43]. These organizations provide substantial financial resources for SRCs. The church at which THS is located provides the premises rent free and pays for maintenance and overheads relating to the facilities which amounts to £ 2056.59 (ZAR 40154.11). The church in providing such assistance is seen as a stakeholder.

### Implications for health and research

Free and charitable healthcare services over time have filled the gap for patients needing medical assistance without the financial means, especially in the United States [44]. Likewise a SRC at the University of New England, Australia, was estimated to save the healthcare system around $437,000.00 in 2013/2014 [45]. This was determined through savings from averting visits to the emergency department. There is insufficient published data on the costs of healthcare in South Africa which could determine the cost savings to the Department of Health. Furthermore, THS provides services to the homeless, many of whom are foreigners and face challenges when accessing public healthcare services [46].

There is no doubt that free clinics provide a much-needed service however, financial sustainability of free clinics is always a challenge and needs innovative cost-effective solutions. Models for developing student-run clinics as sustainable of community-based projects should be considered [24]. Alternate forms of funding of SRCs need to be investigated such as research grants and assistance from universities as well as developing community partnerships [42]. Under the NHI there will be an opportunity to contract with the National Department of Health as a private sector healthcare provider [12] and this study may assist in negotiating the contracting agreement. Furthermore, partnering with the NHI could possible address feasibility concerns facing the SRC.

The homeless community in accessing the services provided by THS has indicated the need for such organizations. The clinic has a responsibility to ensure the continuity of services provided. The services typically focused on acute care as it is unable to provide a full spectrum of services [47]. Thus, it is essential that adequate referral systems are established and these services are integrated within the public healthcare system.

This study provides valuable information to institutions embarking on similar projects, especially with the rising number of SRCs [34]. Furthermore, these findings may, too be of great value in the South African setting as it establishes the NHI.

### Recommendations for further studies

An accurate cost of consumables used in the consultation could not be calculated retrospectively. A system to record this information is essential for accurate stock control as well as budgeting.

The cost of treatment could not be calculated for specific conditions which would allow for comparison in other sites. Implementation of electronic management system where patient data is recorded would assist in documenting the associated costs such as consumables used, and medication prescribed link to a specific diagnosis. Furthermore, the cost of treatment per patient relative to the benefit it brings to the patient should be investigated in future studies.

The development of a clinic formulary helps in the management of stock and assists those prescribing. Further studies could assess the medications included against evidence-based practice.

## Conclusion

This study summarised the costs for establishing and operating a student-run clinic and providing pertinent information essential to the sustainability of the service. The costs of establishing the clinic included building alterations and installations, purchasing of equipment, furniture, consumables and medication as well as licensing of the pharmacy. Rental, personnel, overheads and maintenance costs were the highest expenses contributing to the recurrent costs; however the majority of these are economic costs. The highest financial costs the clinic incurred were overheads, medication, consumables and pharmacy license fees. The results from this study provide a basis of costs which may be of value in the establishment of free clinics in faith-based organisations or universities. In the South African setting where there are limited studies regarding the cost of primary healthcare, it may even have broader application with the emerging national health insurance scheme.

## Data Availability

Available on request (deanne@sitiwi.co.za).

## Abbreviations

£: Pound
NHI: National health insurance
SEP: Single-exit price
SRC: Student-run clinic
THS: Trinity Health Services
ZAR: South African Rands
